# Proanthocyanidin-rich grape seed extract improves bone loss, bone healing, and implant osseointegration in ovariectomized animals

**DOI:** 10.1038/s41598-020-65403-4

**Published:** 2020-06-01

**Authors:** Taichi Tenkumo, Alkebaier Aobulikasimu, Yoshinori Asou, Midori Shirato, Shunichi Shishido, Taro Kanno, Yoshimi Niwano, Keiichi Sasaki, Keisuke Nakamura

**Affiliations:** 1 Division of Advanced Prosthetic Dentistry, Tohoku University Graduate School of Dentistry, 4-1 Seiryo-machi, Aoba-ku, Sendai 980-8575 Japan; 2 Department of Orthopaedics Surgery, Tokyo Medical and Dental University, 1-5-45 Yushima, Bunkyo-ku, Tokyo 113-8510 Japan; 3 Department of Advanced Free Radical Science, Tohoku University Graduate School of Dentistry, 4-1 Seiryo-machi, Aoba-ku, Sendai 980-8575 Japan; 4 Faculty of Nursing, Shumei University, 1-1 Daigaku-cho, Yachiyo, Chiba 276-0003 Japan

**Keywords:** Health care, Dentistry, Bone

## Abstract

The purpose of the present study was to confirm if proanthocyanidin-rich grape seed extract (GSE) had the ability to improve bone health such as bone loss, bone healing, and implant osseointegration (defined as the direct connection between bone tissue and an implant) in ovariectomized (OVX) animals. We demonstrated that daily oral administration of GSE prevented bone loss in the lumbar vertebrae and femur in OVX mice. In addition, osteoclastogenesis in the lumbar spine bone of OVX mice, as assessed by histological and histomorphometric analyses, was accelerated but GSE prevented this dynamization, suggesting that GSE could counteract OVX-induced accelerated osteoclastogenic activity. In rats, OVX clearly impaired the healing of defects created on the calvaria, and GSE overcame this OVX-impaired healing. In the same way, osseointegration of a tibial implant in rats was retarded by OVX, and GSE counteracted the OVX-induced poor osseointegration, likely promoting bone healing by preventing imbalanced bone turnover. These results suggest that orally administered GSE improved implant osseointegration by mitigating the impaired bone health induced by OVX as a model of estrogen deficiency.

## Introduction

There have been epidemiological reports showing that flavonoids, bioactive polyphenols found particularly in fruit and vegetables, ameliorated bone health. A cohort study of females aged between 18 and 79 years (average aged 48 years) demonstrated that total flavonoid intake was positively associated with bone mineral density (BMD), with effects observed at both the hip and spine, supporting a role for flavonoids present in plant-based foods on bone health^1^. A large group study of perimenopausal Scottish women recruited from a population-based screening program for osteoporotic fracture risk showed that associations were found between energy-adjusted total flavonoid intake and improved bone health indicated by BMD at the femoral neck and lumbar spine^2^. Regarding postmenopausal osteoporosis, a common metabolic bone disorder characterized by low BMD and increased fracture risks in postmenopausal women with the cessation in ovarian hormone production^3,4^, a prospective 12-month human intervention study showed that olive polyphenolic compounds may have a promising biological activity in the maintenance of balanced bone turnover in postmenopausal women with osteopenia^5^. These human data suggest that bioactive polyphenols would have beneficial effects on disturbed bone metabolism. Preclinical studies using ovariectomized (OVX) rats, a model for postmenopausal women, and rats fed a low-calcium diet also showed beneficial effects of bioactive polyphenols on bone health, supporting the aforementioned human evidence^6–11^. Of these six preclinical studies, we focused on the study showing that a grape-enriched diet improved calcium utilization and suppressed-bone turnover, resulting in improvements in bone quality in OVX rats^12^, because our recent studies showed that grape seed extract (GSE) rich in proanthocyanidin protected OVX mice from obesity and impaired glucose tolerance^9,10^. Proanthocyanidin is a polymer of flavan-3-ols, a type of flavonoid, with an average range of polymerization between 2 and 17^11,13^, and grape seeds are known to be one of the richest sources of proanthocyanidin^14,15^. Accordingly, GSE is noteworthy for its anti-oxidative activity including scavenging free radicals^16,17^. Besides the anti-oxidative property, it is suggested that GSE has anti-inflammatory, anti-diabetic, anti-obesity, anti-carcinogenic, and anti-ageing effects^18–25^.

If proanthocyanidin-rich GSE improves bone health in aged women with estrogen deficiency, it is likely that it could also be useful in orthopedics and dentistry. Osseointegration, defined as the direct connection between bone tissue and an implant, is used in orthopedics and dentistry^26,27^. Titanium dental implants have been successfully used for decades to treat partial and complete edentulous patients^28–30^, and titanium and titanium-based alloys have also been used for decades as the number one choice for orthopedic implant materials^27,31,32^. The success of dental and orthopedic implant therapy depends on the establishment and maintenance of adequate osseointegration^26^. For instance, pedicle screws that can provide three-column fixation are the most common implants used in spinal surgery, but screw performance depends on bone quality^33–35^. As such, the installation of orthopedic and dental implants may not be an appropriate approach for patients with diseases affecting bone metabolism such as osteoporosis, because of the negative effect of titanium implants on osseointegration^36,37^. Thus, we hypothesized that proanthocyanidin-rich GSE could improve osseointegration in postmenopausal women with osteoporosis, which may in turn enhance the potential for successful integration of implants within the native bone tissue.

The purpose of the present study was to confirm if proanthocyanidin-rich GSE had an ability to prevent bone loss in OVX mice and rats, a model of estrogen deficiency/post-menopausal women, as previously demonstrated in other animal studies^6–8,12,38,39^, and to evaluate the potential effects of proanthocyanidin-rich GSE on the healing of bone defects as well as implant osseointegration in OVX animals.

## Materials and Methods

The present study comprises two parts, described in one sequence: mouse experiments conducted at Tokyo Medical and Dental University and rat experiments conducted at Tohoku University. The common interest of the two research groups, in addition to bone metabolism, is implant osseointegration in orthopedic surgery and dentistry. In order to examine implant osseointegration in relation to bone metabolism *in vivo* we used rats, which seemed much more suitable than mice for implant installation.

### Test substances

GSE (Leucoselect) was obtained from Indena (Milan, Italy). Leucoselect has a well-defined chemical composition elucidated completely by instrumental analyses (e.g., high-performance liquid chromatography-mass spectrometry) by the manufacturer and is> 80% proanthocyanidins. GSE suspended in pure water (PW) was administered by oral gavage to mice at a dose of 100 mg/10 ml/kg/day and to rats at 100 mg/2.5 ml/kg/day. The dose of 100 mg/kg was set according to our previous studies showing that 100 mg/kg exerted positive effects on OVX-induced metabolic disorders in OVX mice^9^.

### Mice and experimental design

**Figure 1:**
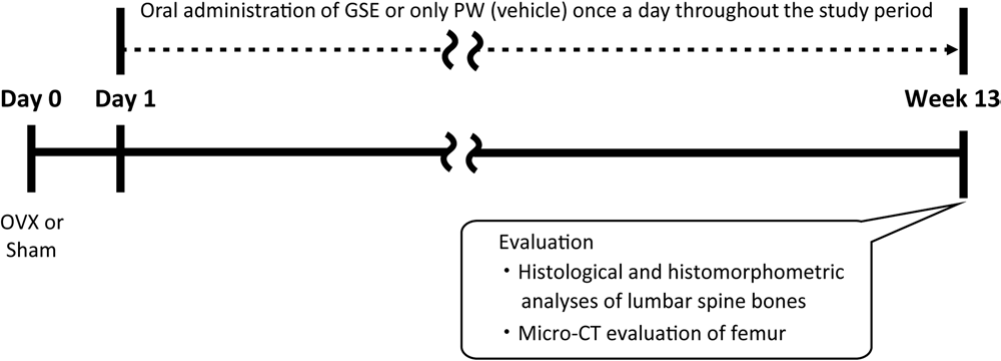
Study design for examining the effect of oral GSE on osteoporosis-like bone loss induced in ovariectomized mice.

All mouse experiments were approved by the Institutional Animal Care and Use Committee of Tokyo Medical and Dental University (approval number: A2019-221C). All institutional and national guidelines for the care and use of laboratory animals were followed. Seven-week-old female C57BL/6 J mice were obtained from Sankyo Labo Service (Tokyo, Japan) and used after acclimatization to their new environment for 1 week. Animals were housed at an approximate temperature and relative humidity of 23–25 °C and 70%, respectively, under a 12/12-h light/dark cycle. Animals that underwent ovariectomy or a sham operation were divided into three groups: (1) Sham operation (Sham) and oral administration of PW as a vehicle (Sham + PW), (2) OVX and oral administration of PW (OVX + PW), and (3) OVX and oral administration of GSE aqueous suspension (OVX + GSE). Ovariectomy was carried out by making two small incisions on the back of each mouse to remove both ovaries under anesthesia with an intraperitoneal injection of 2,2,2-tribromoethanol (Sigma–Aldrich, Saint Louis, MO, USA). The sham operation was carried out in the same manner except that the ovaries were not excised. GSE suspended in PW was administered by oral gavage to the OVX mice at a dose of 100 mg/10 ml/kg/day. The control animals received an equivalent volume of pure water. Oral administration was carried out once daily for 13 weeks from the day following surgical operation. The duration of administration in the present study was consistent with that in our previous study examining the effect of GSE on estrogen deficiency-induced metabolic disorders in OVX mice^9^. Because a positive effect of GSE was shown in the previous study, the same duration was used in the present study. The experimental schedule is shown in Fig. 1.

### Histological and histomorphometric analyses of the lumbar spine bone

The procedures for histological and histomorphometric analyses were essentially identical to those described in a previous study^40^. Calcein (Sigma-Aldrich) was injected subcutaneously at a dose of 25 mg/kg for bone labeling 5 and 2 days before sacrifice, and bone samples of the third and fourth lumbar vertebrae were collected at sacrifice. The samples were immersed in 10% neutral buffered formalin for fixation. Subsequently, they were dehydrated in a series of ethanol solutions and embedded in an acrylic resin and processed for ground sectioning. The undecalcified sections were stained with von Kossa and tartrate-resistant acid phosphatase (TRAP) stains. Static and dynamic histomorphometric analyses were performed using the OsteoMeasure Analysis System (OsteoMetrics, Decatur, GA, USA) following the nomenclature defined by the American Society for Bone and Mineral Research as previously described^41^. Bone volume fraction (the ratio of trabecular bone volume to total volume, BV/TV) was determined using von Kossa-stained specimens according to previous studies^42,43^. On the basis of the TRAP stain, the following five histomorphometric parameters were determined: the number of osteoclasts (N.Oc) per image, the percentage of trabecular bone surface covered by osteoclasts [Oc.S/BS (%)], the N.oc normalized by trabecular bone perimeter [N.Oc/B.Pm (/mm)], the N.oc normalized by osteoclast perimeter [N.Oc/Oc.Pm (/mm)], and the N.Oc normalized by trabecular bone area [N.Oc/T.Ar (/mm^2^)]. In addition, fluorescent microscopy of the trabecular bone section showing the calcein (green) labels was carried out to determine osteogenesis parameters including mineral apposition rate [MAR (µm/day)], bone formation rate per unit of bone surface [BFR/BS (µm^3^/µm^2^/day)], and BFR normalized per unit of bone volume [BFR/BV (%/day)], which is equivalent to the bone turnover rate. These parameters were calculated using the following equations:

$$\begin{array}{ccc}{\rm{MAR}} & = & {\rm{Ir}}{\rm{.L}}{\rm{.Th}}/{\rm{Ir}}{\rm{.L}}{\rm{.t}}\\ {\rm{BFR}}/{\rm{BS}} & = & {\rm{MAR}}({\rm{MS}}/{\rm{BS}})\\ {\rm{BFR}}/{\rm{BV}} & = & {\rm{MAR}}({\rm{MS}}/{\rm{BV}})\end{array}$$

where Ir.L.Th is the inter-label thickness, Ir.L.t is the inter-label time (3 days), MS is the mineralized surface, BS is the bone surface, and BV is the bone volume.

### Micro-computed tomography (CT) evaluation of the femur

The femurs excised from the animals after sacrifice by cervical dislocation were subjected to micro-CT analysis (Comscan, Yokohama, Japan). Two-dimensional images of the distal femurs were obtained by micro-CT, and three-dimensional morphometric parameters were determined using the TRI/3D-Bon software (RATOC, Tokyo, Japan). According to the guidelines for assessment of bone microstructure using micro-CT^44^, the following variables were analyzed for trabecular bone: bone volume fraction [BV/TV (%)], trabecular number [Tb.N (1/mm)], trabecular thickness [Tb.Th (µm)], and trabecular separation [Tb.Sp (µm)]. Similarly, the variables analyzed for cortical bone were as follows: total cross-sectional area [Tt.Ar (mm^2^)], cortical bone area [Ct.Ar (mm^2^)], cortical bone area fraction [Ct.Ar/Tt.Ar (%)], and cortical thickness [Ct.Th (µm^2^)]. The analysis of trabecular bone was performed at a region from 0.2 to 1 mm above the distal growth plates of the femurs while that of cortical bone was done at a mid-shaft region of the femur.

### Rats and experimental design

Female Wistar rats (6 weeks old) were obtained from Charles River Laboratories Japan (Yokohama, Japan). The animals were kept in a plastic cage and housed at a temperature of 19–24 °C under a 12/12-h light/dark cycle. They were given access to standard rodent food pellets (Labo MR Stock, Nosan, Tokyo, Japan) and tap water *ad libitum*. The protocol for the animal experiment was reviewed and approved by the Institutional Animal Experiment Committee of Tohoku University (approval number: 2018DnA-038). All procedures and animal care were performed in accordance with the guidelines adopted by Tohoku University.

**Figure 2:**
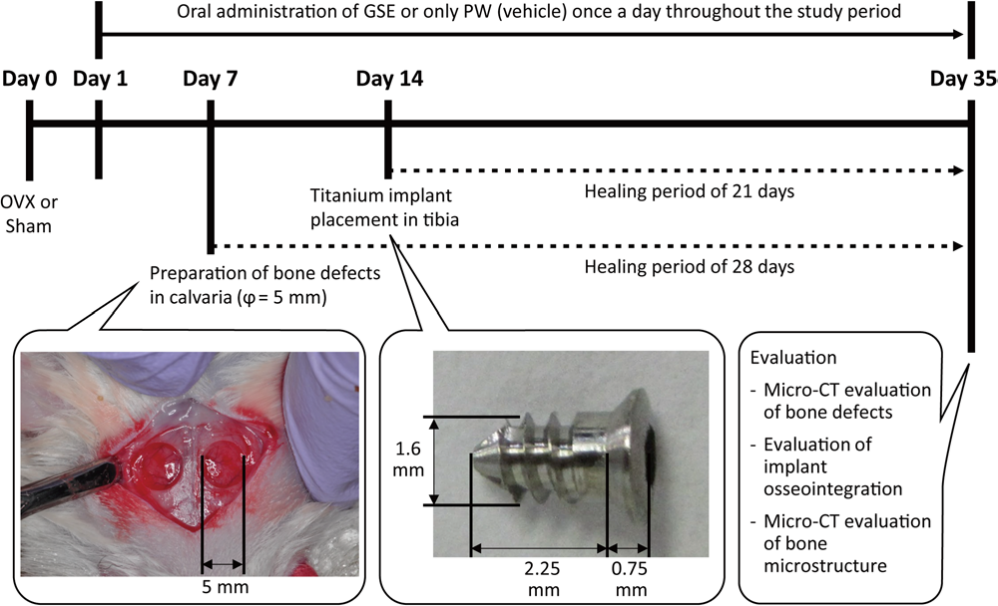
Study design for examining the effect of oral GSE on healing of calvarial bone defects, implant osseointegration in tibia, and femur bone health in ovariectomized rats. OVX: ovariectomy, Sham: sham operation, GSE: grape seed extract.

The study design is illustrated in Fig. 2. After a 1-week acclimatization (Day 0), the animals underwent OVX or a sham operation (Sham). From one day after the surgery (Day 1) to the end of the study period, GSE suspended in PW or only PW was orally administered once a day. Thus, we had following three groups: (1) Sham + PW, (2) OVX + PW, and (3) OVX + GSE. GSE suspended in PW was administered by oral gavage to the OVX rats at 100 mg/2.5 ml/kg. Bone defects were made on the calvaria on Day 7, and titanium implants were installed in the tibia on Day 14. The animals were sacrificed using an overdose of isoflurane inhalation followed by cervical dislocation on Day 35 for assessments of bone healing, implant osseointegration, and bone microstructure. Thus, the bone defects and the tissues around the implants were allowed to heal for 28 and 21 days, respectively. In addition, the effect of GSE on the bone healing in intact rats was examined. PW or GSE were similarly administered once a day throughout the experiment.

### Surgical procedures

All surgical procedures were performed under anesthesia with a subcutaneous injection of 0.15 mg/kg medetomidine (Nippon Zenyaku Kogyo, Koriyama, Japan), 2 mg/kg midazolam (Sandoz, Tokyo, Japan), and 2.5 mg/kg butorphanol tartrate (Meiji Seika Pharma, Tokyo, Japan). For OVX and sham operations, two small incisions were made in the rats’ backs to pull out bilateral ovaries using ring tweezers. The ovaries of the animals in the OVX group were excised and removed while those in the sham group were returned to the original position. The uterine weight of the animals was measured to confirm the effect of ovariectomy at Day 35.

Bone defects with a diameter of 5 mm were created on the calvaria of the animals. In order to examine the effect of GSE on bone healing in intact animals, bone defects were also created in intact animals. A sagittal incision was made on the head after shaving, followed by raising a full-thickness periosteal flap. Two circular osseous defects were prepared according to previous studies^45,46^. Briefly, a trephine bur with an external diameter of 5 mm was operated at 6000 rpm with sterile saline irrigation using a dental electric motor system (VIVAace, NSK, Kanuma, Japan). The operation using a trephine bur was stopped before complete penetration to avoid damage to the brain. The circular bone within the cutting groove was removed using a small excavator. Subsequently, the periosteum and skin were separately sutured.

Screw-shaped titanium implants were installed into the proximal metaphysis of the tibias of the animals. A small incision was made on the internal side of knee joints after shaving, and the recipient sites of the implants were prepared using a drill (diameter = 1.5 mm) operated using the dental electric motor system. Then, a small titanium screw (Fig. 2; c.p. titanium M 1.6 screw, Fukuoka Seimitu, Sabae, Japan) with a machined surface was installed with the self-tapping technique. Subsequently, the wound was closed with multiple simple sutures.

### Micro-CT evaluation of bone defects

The calvaria was removed from the animal after sacrifice. Bone healing in 12 defects per group was evaluated by measurement of the volume of bone defects left on the calvaria using a micro-CT (ScanX-mate-E090, Comscantecno, Kanagawa, Japan). The measurement conditions were as follows: voltage, 60 kV; current, 80 µA; resolution (voxel size), 50.0 µm; and 600 projections. The CT data were reconstructed using a software package (Cone CT Express, Comscantecno), and the reconstructed images were analyzed to determine the volume of bone defects using ImageJ, an image-processing program, provided by the Research Services Branch of the National Institutes of Health (Bethesda, MD, USA).

### Evaluation of implant osseointegration

Biomechanical analysis of implant osseointegration was performed by the removal torque test. Immediately after euthanasia, the skin at the implant site was cut and opened, and the removal torque of nine titanium screws (implants) per group was measured using a digital torque tool (GLK060, KTC, Kyoto, Japan). The maximum loosening torque of the screw was recorded in N⋅cm.

Three implants with the surrounding tissues in each group, which were not subjected to the removal torque test, were removed for histological evaluation. The tissue block was immersed in 10% neutral buffered formalin for fixation. Subsequently, the samples were dehydrated in a series of ethanol solutions and embedded in methyl methacrylate resin (Wako Pure Chemicals Industries, Osaka, Japan). Undecalcified ground sections were prepared using a micro cutting machine (BS-300CP, Exact, Hamburg, Germany) and a micro grinding machine (MG-400CS, Exact). A central section of each implant was ground and polished to a 40 µm thickness followed by staining with hematoxylin and eosin for microscopy. Bone-to-implant contact (BIC) percentage was evaluated along the entire implant periphery installed in the bone on the histological images using ImageJ.

### Micro-CT evaluation of bone microstructure

The influence of proanthocyanidin-rich GSE administration on bone density in OVX rats was evaluated in the femur of the animal. The femurs were removed from the animals in each group, and six femurs per group were subjected to micro-CT analysis. The measurement conditions were generally the same as described above except the resolution was set to 67 µm because of the size of the femurs. As in the case of the mouse experiment described above, BV/TV (%), Tb.N (1/mm), Tb.Th (µm), Tb.Sp (µm), Tt.Ar (mm^2^), Ct.Ar (mm^2^), Ct.Ar/Tt.Ar (%), and Ct.Th (µm^2^) were measured. The analysis was performed in a 3-mm thick region of interest and 1.5 mm away from the reference line defined by both ends of epiphyseal growth plate using a software package (TRI/3D-Bon).

### Statistical analyses

The data obtained were subjected to statistical analyses using JMP Pro 13.1 software (SAS Institute, Cary, NC, USA). Statistical analyses for the quantitative measures between the three groups were performed using the Tukey–Kramer multiple-comparison test. To determine the significant difference between the two groups, Student’s *t*-test was applied following the two-sided F-test.

## Results

### Mouse experiments

#### Histological and histomorphometric analyses of lumbar spine bone

**Figure 3:**
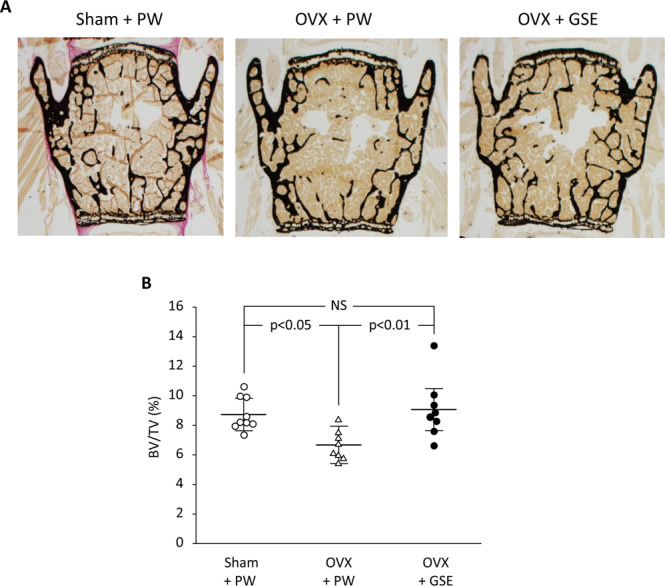
Effect of oral administration of proanthocyanidin-rich grape seed extract (GSE) on bone loss in the third and fourth lumbar vertebrae from spines of ovariectomized (OVX) mice. (**A**) Representative images of von Kossa-stained third vertebrae from each of the three groups. (**B**) Trabecular bone volume/total volume (BV/TV) ratio was determined by histological analysis of lumbar spine bones. In B, the results are expressed the mean ± standard deviation, showing individual data (n = 9 in Sham + PW, n = 8 in OVX + PW and OVX + GSE). PW: pure water, NS: not significant (p > 0.05).

Representative microscopic images of the lumbar spine from each of the three groups are shown in Fig. 3A. Histological analysis showed that BV/TV in the vertebrae was significantly lower (p < 0.05) in OVX mice than in sham mice (Fig. 3B).

**Figure 4:**
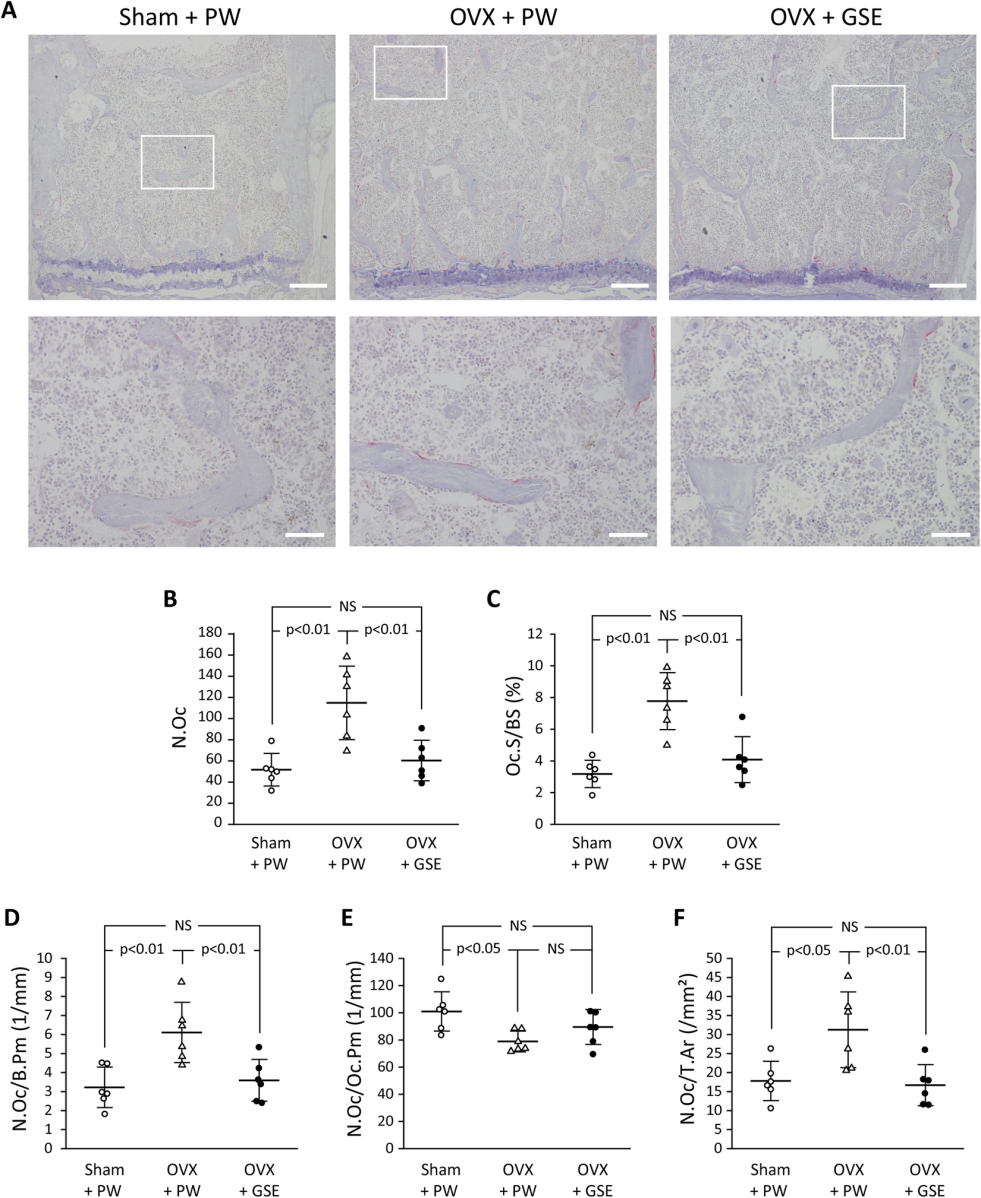
Histological and histomorphometric evaluation of osteoclastogenic parameters in the third and fourth lumbar vertebrae of ovariectomized (OVX) mice administered with proanthocyanidin-rich grape seed extract (GSE). (**A**) Representative images of TRAP-stained sections of lumbar vertebrae from each of the three groups. The area under the white square in the top row was analyzed at higher magnification as shown in the bottom row. Scale bars in the top and bottom row represent 200 and 50 µm, respectively. (**B**–**F**) Quantification of osteoclast-associated histological measures. In (**B**–**F**), the results are expressed as the mean ± standard deviation, showing individual data (n = 6). N.Oc: the number of osteoclasts, Oc.S/BS: the ratio of osteoclast surface to trabecular bone surface, N.Oc/B.Pm: the ratio of N.Oc to bone perimeter, N.Oc/Oc.Pm: the ratio of N.Oc to osteoclast perimeter, and N.Oc/T.Ar: the ratio of N.Oc to trabecular bone area. NS: not significant (p > 0.05).

**Figure 5:**
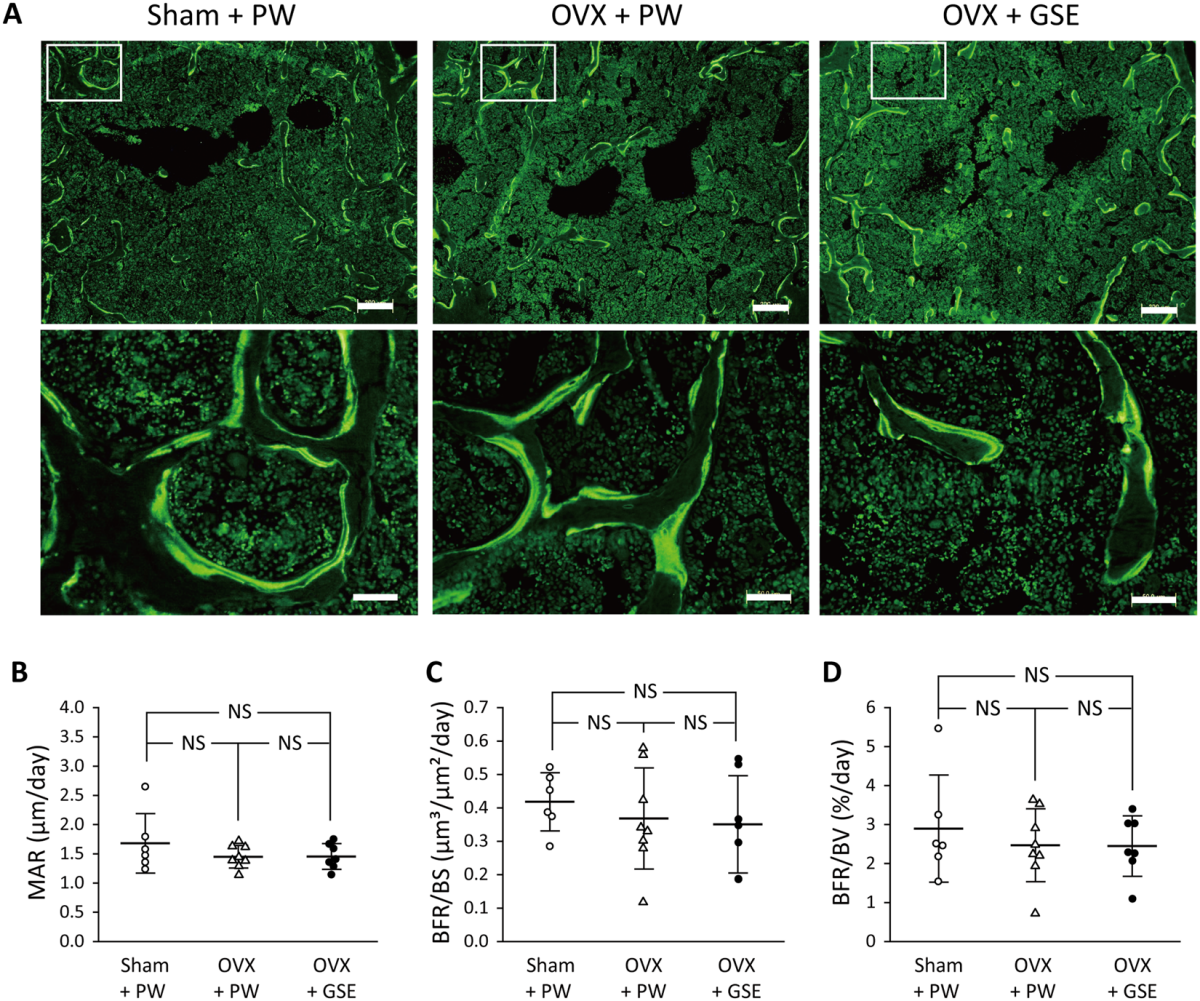
Histological and histomorphometric evaluation of osteogenic parameters in the trabecular bone in ovariectomized (OVX) mice administered with proanthocyanidin-rich grape seed extract (GSE). (**A**) Representative fluorescent microscopy images showing the calcein (green) labels of the trabecular bone sections of lumbar vertebrae from each of the three groups. The area under the white square in the top row was analyzed at higher magnification as shown in the bottom row. Scale bars in the top and bottom row represent 200 and 50 µm, respectively. (**B**–**D**) Quantification of osteogenesis-associated histological measures. In (**B**–**D**), the results are expressed as the mean ± standard deviation, showing individual data (n = 6 in Sham + PW, n = 8 in OVX + PW, n = 7 in OVX + GSE). MAR: mineral apposition rate, BFR/BS: bone formation rate/bone surface, and BFR/BV: BFR/bone volume. No significant differences in any of the three measures were found between the three groups.

Oral administration of GSE to OVX mice kept the BV/TV level similar to that in the sham group (p > 0.05). Representative microscopic images of TRAP-stained sections of the lumbar spine from each of the three groups are shown in Fig. 4A. Bone histological analysis of the lumbar spine also showed that all the five osteoclast-related measures (N.Oc, Oc.S/BS, N.Oc/B.Pm, N.Oc/Oc.Pm, and N.Oc/T.Ar) were significantly affected by OVX compared to those in the sham group, and oral administration of GSE to OVX mice prevented such changes in the OVX group except for N.Oc/Oc.P (Fig. 4B–F). Although N.Oc/Oc.Pm in OVX + PW was significantly lower than that in Sham + PW (p < 0.05), a significant difference was not found between OVX + PW and OVX + GSE. There was also no significant difference in N.Oc/Oc.Pm between Sham + PW and OVX + GSE. For the four measures other than N.Oc/Oc.Pm, OVX + PW > Sham + PW ≈ OVX + GSE for N.Oc (Fig. 4B), Oc.S/BS (Fig. 4C), N.Oc/B.Pm (Fig. 4D), and N.Oc/T.Ar (Fig. 4E). Representative images of fluorescent microscopy of the calcein (green) labeled trabecular bone sections of the lumbar vertebrae are shown in Fig. 5A, and three osteogenesis-related measures (MAR, BFR/BS, and BFR/BV) are summarized in Fig. 5B–D, respectively. In contrast to the osteoclast-related measures, no significant differences in any of the osteogenesis-related measures were found between the Sham + PW, OVX + PW, and OVX + GSE groups.

#### Micro-computed tomography (CT) evaluation of the femur

**Figure 6:**
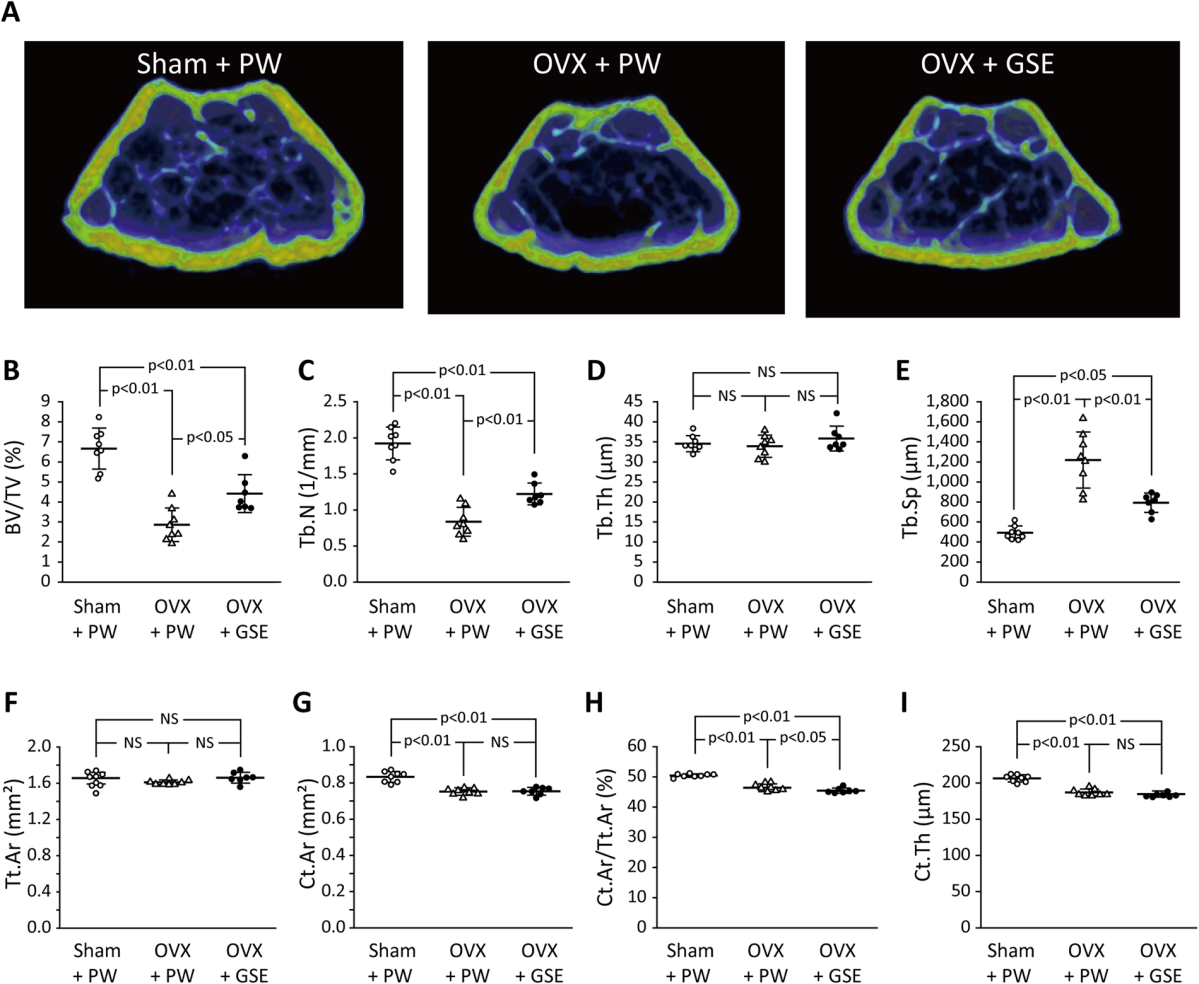
Effect of oral administration of proanthocyanidin-rich grape seed extract (GSE) on bone loss at the distal femur region in ovariectomized (OVX) mice. (**A**) Representative micro-CT images of the distal femur region from each of the three groups. (**B**–**I**) Micro-CT variables of trabecular and cortical bone determined for each group. BV/TV: trabecular bone volume fraction, Tb.N: trabecular number, Tb.Th: trabecular thickness, Tb.Sp: trabecular separation, Tt.Ar: total cross-sectional area, Ct.Ar: cortical bone area, Ct.Ar/Tt.Ar: cortical bone area fraction, Ct.Th: cortical thickness. In (**B**–**I**), the results are expressed as the mean ± standard deviation, showing individual data (n = 8 in Sham + PW and OVX + PW, n = 7 in OVX + GSE).

Representative micro-CT images of the distal femur region from each of the three groups are shown in Fig. 6A. As was the case with the histological analysis of the vertebrae, micro-CT analysis revealed that BV/TV in the femurs was significantly lower (p < 0.01) in OVX mice than in sham mice (Fig. 6B), and oral administration of GSE to OVX mice prevented the BV/TV level from lowering (p < 0.05 between OVX + PW and OVX + GSE) even though the level was still significantly lower than that in the sham group (p < 0.01) (Fig. 6B). OVX also significantly decreased and increased Tb.N and Tb.Sp, respectively, while Tb.Th was not significantly affected (Fig. 6C–E). Oral administration of GSE tended to improve these parameters of trabecular bone microstructure, resulting in significantly higher Tb.N and Tb.Sp in OVX + GSE than in OVX + PW (Fig. 6C,E). Regarding cortical bone (Fig. 6F–I), the OVX significantly decreased Ct.Ar, Ct.Ar/Tt.Ar, and Ct.Th (p < 0.01), though the differences were small. The influence of oral administration of GSE on the cortical bone of OVX mice was limited. Consequently, the differences in Ct.Ar and Ct.Th between OVX + PW and OVX + GSE were not significant except that the OVX + GSE group showed significantly lower Ct.Ar/Tt.Ar than the OVX + PW group (Fig. 6F–I).

### Rat experiments

#### Uterine weight

**Figure 7:**
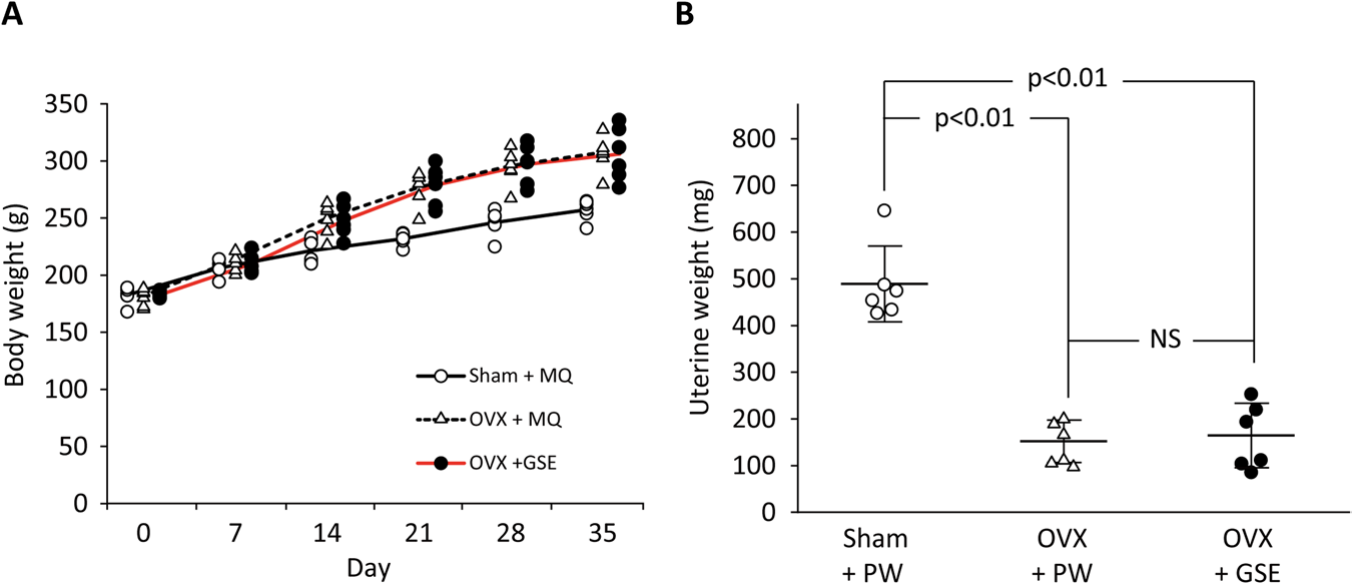
Effect of oral administration of proanthocyanidin-rich grape seed extract (GSE) on body weight and uterine weight in ovariectomized (OVX) rats. (**A**) The time-course changes in body weight. The results are expressed as the mean, showing individual data (n = 6). (**B**) Uterine weight measured at Day 35. The results are expressed as the mean ± standard deviation, showing individual data (n = 6). NS: not significant (p > 0.05).

To confirm the effects of the ovariectomy, rat uterine weight was measured at the time of sacrifice (Fig. 7). The OVX + PW group showed significantly lower uterine weight than the sham group (p < 0.01), confirming uterine atrophy due to the ovariectomy (Fig. 7B). The weight of the OVX + GSE group was similar to that of the OVX + PW group (Fig. 7A). Relative uterine weight to body weight confirmed that the uterine atrophy was clearly induced by OVX and GSE did not affect it.

#### Micro-CT evaluation on bone defects

**Figure 8:**
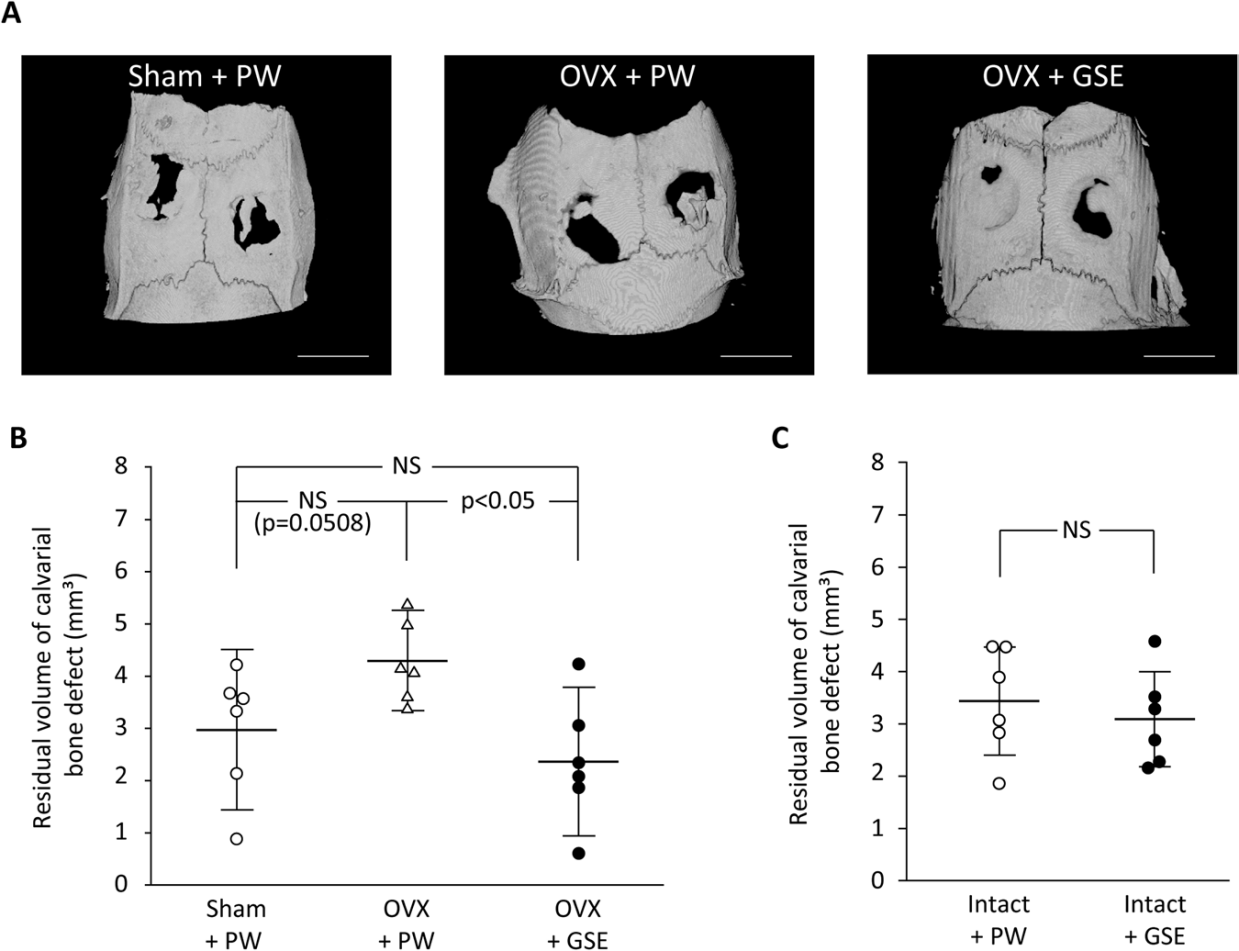
Effect of oral administration of proanthocyanidin-rich grape seed extract (GSE) on bone healing in ovariectomized (OVX) and intact rats. (**A**) Representative micro-CT images of bone defects on the calvaria in Sham + PW, OVX + PW, and OVX + GSE groups 28 days after defect creation. Scale bar = 5 mm. (**B**) Quantification of bone defect volume based on 3D-reconstructed micro-CT images obtained from the OVX animals. Oral administration of GSE resulted in significantly lower volume of defects than did administration of pure water (PW) in OVX rats, whereas GSE did not significantly affect bone healing in the sham-operated rats (Sham). (**C**) Quantification of bone defect volume based on 3D-reconstructed micro-CT images obtained from the intact animals. No significant difference was found between the intact + PW and intact + GSE groups. In (**B**,**C**), the results are expressed as the mean ± standard deviation, showing individual data (n = 6).

Representative micro-CT images of the bone defects in the Sham + PW, OVX + PW, and OVX + GSE groups after a 28-day healing period are shown in Fig. 8A. New bone was formed from the periphery of the bone defects irrespective of the group. However, in the OVX + PW group, bone defects were still relatively large in comparison with those of the other two groups, indicating that bone healing in the OVX + PW group was deterred. Consequently, the quantitative analysis of the defect volume based on the reconstructed micro-CT images showed that the volume in the OVX + GSE group was significantly smaller than that in OVX + PW (p < 0.05, Fig. 8B). On the contrary, there was no significant difference in the volume of bone defects between the intact + PW and intact + GSE groups (Fig. 8C).

#### Evaluation of implant osseointegration

Three randomly selected implants from each of the three groups were subjected to histological analysis, and the remaining nine implants in each group were used for a removal torque test.

**Figure 9:**
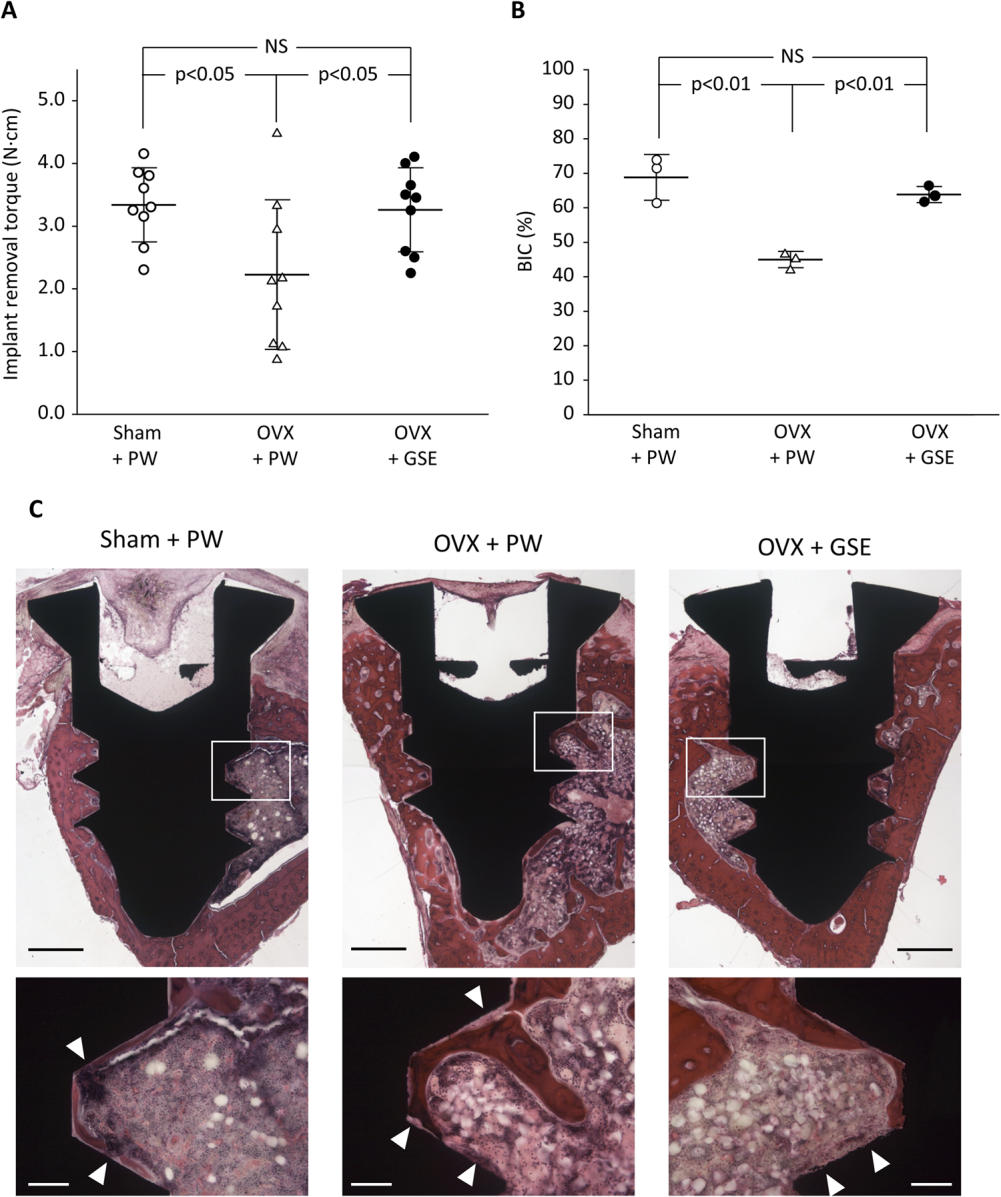
Biomechanical and histological evaluation of implant osseointegration in ovariectomized (OVX) rats administered with proanthocyanidin-rich grape seed extract (GSE). (**A**) Removal torque (N⋅cm) of titanium screw-shaped implant installed in the tibia. Oral administration of GSE resulted in a significantly higher removal torque than did administration of pure water (PW) in OVX rats, whereas GSE did not significantly affect the implant removal torque in sham-operated (Sham) rats. The results are expressed as the mean ± standard deviation, showing individual data (n = 9). (**B**) Quantification of bone-to-implant contact (BIC) percentage in each group. The results are expressed as the mean ± standard deviation, showing individual data (n = 3). (**C**) Representative histological images of implants in Sham + PW, OVX + PW, and OVX + GSE groups obtained at low and high magnifications. The low magnification images are composite views generated based on the images obtained using a 5× objective lens. The high magnification images were obtained using a 20× objective lens, which correspond to the white square area in the low magnification images. White triangles in the high magnification images indicate the part where bone does not directly contact the implant surface. Scale bars in the low and high magnification images represent 500 and 100 µm, respectively.

The mean implant removal torque for the Sham + PW, OVX + PW, and OVX + GSE groups were 3.34, 2.23, and 3.26 N⋅cm, respectively (Fig. 9A). Significant differences were obtained between the Sham + PW and OVX + PW groups (p < 0.05) and between the OVX + PW and OVX + GSE groups (p < 0.05), but not between the Sham + PW and OVX + GSE groups (p > 0.05).

Results of histological analyses are shown in Fig. 9B and 9C. The direct contact of bone with the titanium screw (i.e., osseointegration) as well as new bone formation in the area between the threads were observed in all three groups (Fig. 9C). However, in the OVX + PW group, the new bone was found to less frequently contact the implant directly compared to that in the other two groups when observed at high magnification (Fig. 9C). Consequently, the BIC percentages evaluated based on the histological images were 68.8%, 45.0%, and 63.8% for the Sham + PW, OVX + PW, and OVX + GSE groups, respectively (Fig. 9B). Significant differences were obtained between the Sham + PW and OVX + PW groups (p < 0.01) and between the OVX + PW and OVX + GSE groups (p < 0.01), but not between the Sham + PW and OVX + GSE groups (p > 0.05).

#### Micro-CT evaluation of the femur

**Figure 10:**
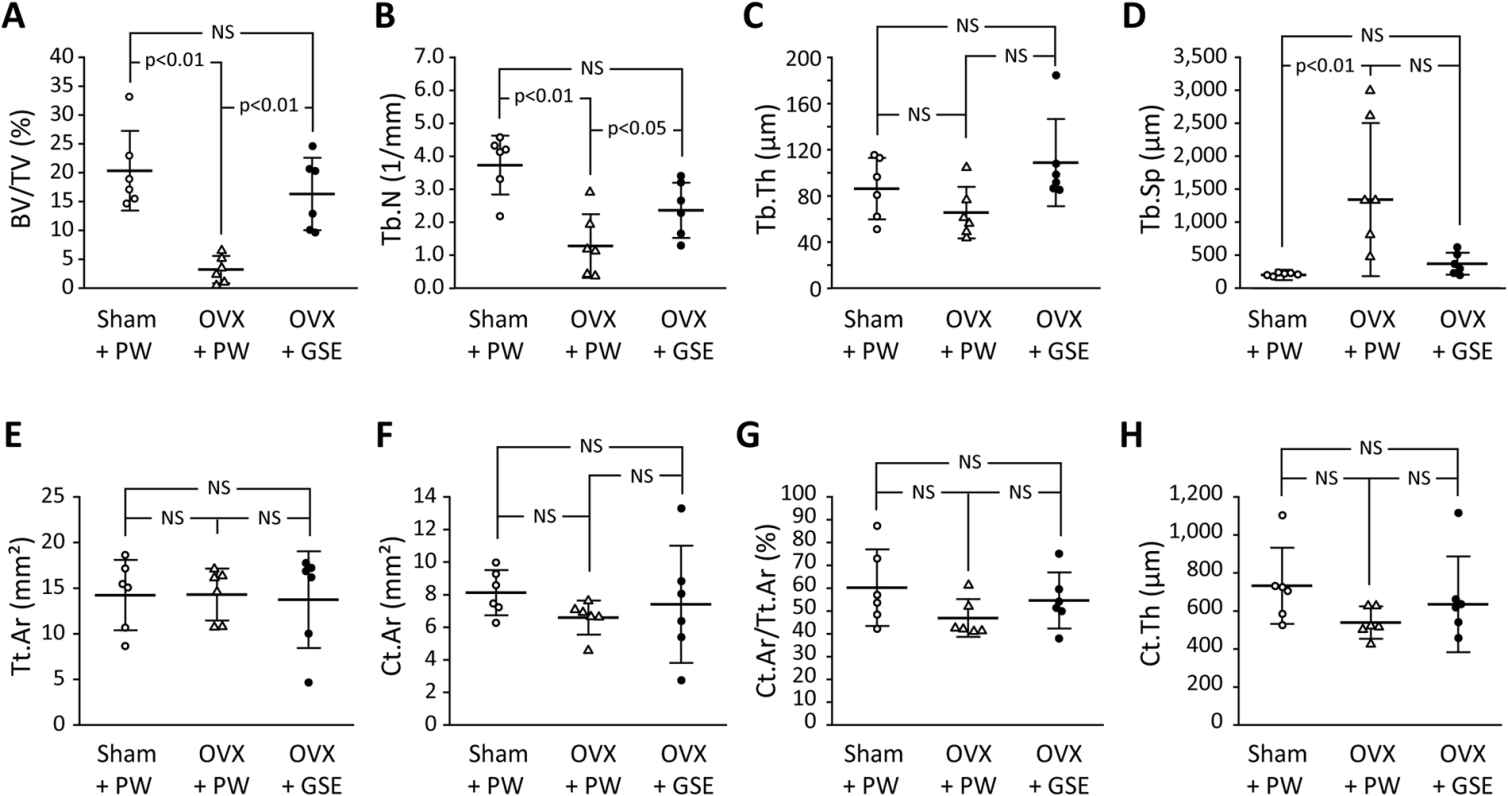
Effect of oral administration of proanthocyanidin-rich grape seed extract (GSE) on trabecular and cortical bone of ovariectomized (OVX) rats. BV/TV: trabecular bone volume fraction, Tb.N: trabecular number, Tb.Th: trabecular thickness, Tb.Sp: trabecular separation, Tt.Ar: total cross-sectional area, Ct.Ar: cortical bone area, Ct.Ar/Tt.Ar: cortical bone area fraction, Ct.Th: cortical thickness. The results are expressed as the means and standard deviations, showing individual data (n = 6).

As shown in Fig. 10A, micro-CT analysis revealed that the BV/TV in the OVX + PW group was about 3% whereas that in the Sham + PW group was over 20%. Oral administration of proanthocyanidin-rich GSE improved the bone density in OVX rats, resulting in a significantly higher BV/TV (16.3%) than PW administration did (p < 0.01). OVX also significantly decreased and increased Tb.N and Tb.Sp, respectively, while Tb.Th was not significantly affected (Fig. 10B–D). The oral administration of GSE tended to improve these parameters of trabecular bone microstructure, resulting in significantly higher Tb.N in OVX + GSE than in OVX + PW (Fig. 10B). Additionally, Tb.Sp in OVX + GSE was comparable to that in Sham + PW. In contrast, the influences of OVX and oral administration of GSE on cortical bone were limited. Consequently, no significant differences in any parameter of cortical bone morphology were detected between the groups (Fig. 10E–H).

## Discussion

In the present study, we demonstrated that daily oral administration of proanthocyanidin-rich GSE not only prevented bone loss of the lumbar spine and femur in OVX mice but facilitated bone healing and implant osseointegration in OVX rats. According to the micro-CT analyses in both mice and rats, OVX affected trabecular bone rather than cortical bone, and GSE administration attenuated the trabecular bone loss caused by OVX. Although OVX resulted in significantly lower Ct.Ar, Ct.Ar/Tt.Ar, and Ct.Th in mice, the differences were rather small as previously reported^47^, and as such, the effect of GSE on cortical bone was not clearly observed.

In the mouse experiment, osteoclastogenesis assessed by histological and histomorphometric analyses in TRAP-stained sections (i.e., N.Oc, Oc.S/BS, N.Oc/B.Pm, and N.Oc/T.Ar, all of which reflect the number and/or size of osteoclasts) increased in the lumbar spine bone in OVX mice as also seen in estrogen-deficient aged women^48,49^, and GSE prevented this dynamization. In contrast to these osteoclastogenesis-related histomorphometric measures, osteogenesis-related histomorphometric measures of the trabecular bone sections of lumbar vertebrae (i.e., MAR, BFR/BS, and BFR/BV, all of which reflect bone formation) did not show any drastic differences between the three groups. This indicated that neither OVX nor OVX + GSE affected osteogenic activity in the trabecular bone under the present experimental conditions. Thus, the bone loss observed in OVX animals was likely due to accelerated osteoclastogenic activity induced by the estrogen deficiency as indicated by a review article suggesting that the deficiency leads to the imbalance of bone turnover in which resorption outpaces formation^50^. As such, GSE would counteract the accelerated osteoclastogenic activity, resulting in the prevention of this imbalanced bone turnover.

In the rat experiment, OVX tended to impair bone healing of the defects created on the calvaria (p = 0.0508 between Sham + PW and OVX + PW), and GSE counteracted the OVX-induced impairment in bone healing. However, as in the mice, GSE did not seem to enhance osteogenic activity, since GSE did not affect bone healing in the calvarial defects of the intact rats. Analogous to the case of calvarial bone healing, GSE increased the implant removal torque and BIC in OVX rats. Osseointegration, which is established as a result of bone healing via inflammation, angiogenesis, osteogenesis, and remodeling^51^, was negatively affected by OVX, resulting in a lower implant removal torque and lower BIC. GSE was able to counteract the lowered osseointegration induced by OVX. The impaired osseointegration induced by OVX was also in accordance with previous findings^52–55^. In addition, previous studies^8,12,56–58^ showed beneficial effects of GSE on bone healing or formation under certain pathological conditions, such as OVX-induced osteoporosis, osteoarthritis, or bone debility induced by a low-calcium diet. As for the mechanisms by which GSE attenuated the OVX-induced impaired bone healing and implant osseointegration, GSE may not mimic estrogen effects because GSE did not attenuate OVX-induced uterine atrophy.

From the results of the present study in mouse and rat, one possible mechanism by which GSE acts on bone health in OVX animals is by affecting estrogen receptors. It was reported that polyphenols such as resveratrol, kaempferol, anthocyanins, and quercetin would be phytoestrogens^59–63^. However, as aforementioned, GSE may not mimic the effects of estrogen. Another possible mechanism is that the preventive effect of GSE on bone loss observed in OVX animals may be due to its anti-oxidative and anti-inflammatory properties. Indeed, many *in vitro* cell culture studies showed that proanthocyanidins^58^ and catechins^64^, in addition to resveratrol^65^, quercetin^66^, and kaempferol^58^, can directly affect bone cells by lowering oxidative stress and inflammation. However, regarding these possible mechanisms, the poor bioavailability of orally administered proanthocyanidins would be an obstacle^67^. That is, a large proportion of dietary proanthocyanidin is not absorbed in the small intestine and reaches the colon^14,68^. Thus, it is questionable if such bioactivities demonstrated by *in vitro* studies play a significant role in the *in vivo* therapeutic effects. Instead, recent studies suggest that proanthocyanidin-rich GSE may partly exert its therapeutic effects via modification of the intestinal microflora^10,38^, which is closely related to various immunological and pathological processes^69,70^. In our study^10^, the Firmicutes/Bacteroidetes ratio (F/B ratio) increased in OVX mice compared with sham mice; however, oral GSE intake decreased the F/B ratio in OVX mice. Moreover, Wang *et al*.^71^ reported that F/B ratios increased in osteoporosis patients (3.326) and osteopenia patients (1.755) compared with those of normal controls (1.290). Given these results from previous studies, the positive effect of oral administration of proanthocyanidin-rich GSE on bone healing and osseointegration found in the present study may be exerted by prevention of the change in intestinal microbiota caused by OVX. However, even if the intestinal microbiota is involved in the positive effect of GSE on bone health, it is still unclear how the microbiota influences bone metabolism. The mechanism of action of proanthocyanidin-rich GSE remains as the major challenge, and should be elucidated in future studies.

Osteoporosis therapies with hormone replacement and bisphosphonates are effective to reduce fracture risk^72^. However, there are concerns regarding an increased risk of severe adverse events of these therapies, such as certain cancers, heart disease, and osteonecrosis of the jaw^73–75^. Thus, as a safe complementary alternative not only for osteoporosis itself but for orthopedic and dental implant treatment in osteoporosis patients, we investigated the potential effects of oral proanthocyanidin-rich GSE, which may not induce local and systemic toxicological effects^76^, on bone health in relation to implant osseointegration in the present study. However, our study has some limitations. In the rat experiments, because the preventive effect of GSE on the OVX-induced impaired bone healing was the main focus, detailed information on how GSE affects the imbalance of bone turnover is lacking. Thus, to fully understand the effects of GSE on bone health, the relationship between bone formation and bone resorption should be examined by applying histomorphometric analysis in the near future. In addition, we implanted titanium screws in the tibia of rats instead of in the alveolar bone to simplify the surgical procedures but due to this, we lack information on the direct potential effects on dental implants. As the biological reactions in bones as well as stress generation on a titanium implant generated by normal physical activity are not identical in the tibia and alveolar bones^51^, the process of osseointegration may also be different. In addition, the observation period was relatively short (i.e., 21 days after implant surgery), and as such future studies need to verify if the beneficial effects on implant osseointegration found in the present study are long-lasting.

Within the limitations of the present study, we conclude that orally administered proanthocyanidin-rich GSE can prevent bone loss and degradation of the implant osseointegration induced by OVX as a model of estrogen deficiency. Further pre-clinical and clinical studies are necessary to validate the findings of the present study.

## Data Availability

The datasets generated and/or analyzed during the current study are available from the corresponding authors upon reasonable request.
